# A specific scoliosis classification correlating with brace treatment: description and reliability

**DOI:** 10.1186/1748-7161-5-1

**Published:** 2010-01-27

**Authors:** Manuel D Rigo, Mónica Villagrasa, Dino Gallo

**Affiliations:** 1 Institut Elena Salvá, Vía Augusta 185, 08021 Barcelona, Spain; 2 Ortholutions, Königsseestrasse 10, D-83022 Rosenheim, Germany

## Abstract

**Background:**

Spinal classification systems for scoliosis which were developed to correlate with surgical treatment historically have been used in brace treatment as well. Previously, there had not been a scoliosis classification system developed specifically to correlate with brace design and treatment. The purpose of this study is to show the intra- and inter- observer reliability of a new scoliosis classification system correlating with brace treatment.

**Methods:**

An original classification system ("Rigo Classification") was developed in order to define specific principles of correction required for efficacious brace design and fabrication. The classification includes radiological as well as clinical criteria. The radiological criteria are utilized to differentiate five basic types of curvatures including: (I) imbalanced thoracic (or three curves pattern), (II) true double (or four curve pattern), (III) balanced thoracic and false double (non 3 non 4), (IV) single lumbar and (V) single thoracolumbar. In addition to the radiological criteria, the Rigo Classification incorporates the curve pattern according to SRS terminology, the balance/imbalance at the transitional point, and L4-5 counter-tilting. To test the intra-and inter-observer reliability of the Rigo Classification, three observers (1 MD, 1 PT and 1 CPO) measured (and one of them, the MD, re-measured) 51 AP radiographs including all curvature types.

**Results:**

The intra-observer Kappa value was 0.87 (acceptance >0.70). The inter-observer Kappa values fluctuated from 0.61 to 0.81 with an average of 0.71 (acceptance > 0.70).

**Conclusions:**

A specific scoliosis classification which correlates with brace treatment has been proposed with an acceptable intra-and inter-observer reliability.

## Background

Idiopathic scoliosis is a multi-factorial, three-dimensional deformity of the spine and the trunk which can appear and sometimes progress during any of the rapid periods of growth in apparently healthy children. Although the three-dimensional nature of the deformity has been recognized for a long time, lateral deviation in the frontal plane has been considered the main radiological diagnostic sign. The assessment of the Cobb angle is essential for diagnosis, follow up and evaluation of treatment results [1]. Although some data suggest that vertebral deformity is already present in scoliosis under 10° [2], the Scoliosis Research Society (SRS) continues to define idiopathic scoliosis as a lateral deviation of the spine measuring 10° Cobb or more with a certain amount of rotation. Progression is defined as an increase of the Cobb angle over a particular period of time. The importance of frontal plane projection cannot be denied, although concentrating solely on this one-dimensional view of a complex scoliotic 3-D geometry may cause serious errors in diagnosis and subsequent treatment of IS [3]. For many decades a constant effort has been made to classify the curve patterns in this frontal projection for a plethora of reasons: to describe the deformity, to predict its spontaneous evolution, to implement a proper treatment plan, to establish the correct surgical strategy, to define the biomechanical principles of the brace and to select curve-specific exercises. In 1950, Ponseti and Friedman published a study on 394 untreated patients with idiopathic scoliosis with different curve patterns. They concluded that these anatomic-radiological forms showed differentiated types of evolutions, pathological consequences and therapy approaches [4]. Moe and Kettleson [5] recognized three single curve types: thoracic, thoracolumbar and lumbar; and four combined curve types: main thoracic/minor lumbar, double major thoracic/lumbar, double major thoracic/thoracolumbar and thoracic double major. Lonstein and collaborators later introduced a single upper thoracic type and analysed the behaviour of the upper structural curve, thereby creating a new concept of the double thoracic curve pattern [6]. Moe and Kettleson's classification system was generally the most commonly used by orthopaedic surgeons and rehabilitation doctors until the introduction of the King classification [7]. The King Classification enjoyed widespread acceptance and is still used in brace design [8]. However, Cummings and collaborators showed that the King Classification had a poor reliability [9]. Furthermore, Lenke and collaborators also concluded that the King Classification does not appear to have sufficient interobserver and intraobserver reliability among scoliosis surgeons to enable accurate curve pattern delineation [10]. In 2001, Lenke presented a new classification to determine the extent of spinal arthrodesis [11]. The Lenke Classification has been widely used since then and reliability has been shown to be better than the King classification in some studies [12]. Additionally, the Lenke classification correlates well with the treatment plan when surgery is the treatment [13,14]. The Lenke Classification is less appropriate for brace design. Historically, brace design has been based on a single classification differentiating between single and double curve patterns. In 2001, d'Amato and collaborators published a paper presenting the results of nighttime bracing with the Providence brace in adolescent girls with IS [15], where brace design was based on a simple classification. The Providence brace system proposes three basic models: lumbar, thoracolumbar and double curve brace designs, with an extension available for high thoracic curves. This simplified approach had been used previously by Lehnert-Schroth [16] to differentiate two functional types of curves in physical therapy, for which she developed the nomenclature 'three curves scoliosis pattern' and 'four curves scoliosis pattern'. The terms and diagnosis criteria defined by Lehnert-Schroth appeared simple but, were, in fact more sophisticated than a mere classification of single and double. She used the terms 'three curve pattern' and 'four curve pattern' to differentiate between single thoracic with no lumbar or with a minor lumbar curve ('three curves scoliosis pattern') from a true double curve associated with a compensatory-lumbosacral curve ('four curve scoliosis pattern'). In addition, Lehnert-Schroth had categories for single lumbar and thoracolumbar scoliosis. Later, Chêneau incorporated Lehnert-Schroth's three and four curve scoliosis pattern terminology but not the Schroth criteria [17]. Chêneau initially defined 'three curve scoliosis' as any single curve and 'four curve scoliosis' as any double curve; correspondingly, he proposed two basic brace designs also called 'three curve scoliosis brace' and 'four curve scoliosis brace'.

Since 1968, the protocol of the Barcelona School of Scoliosis Rehabilitation ("BSSR") is supported by specific three-dimensional physical therapy methods and bracing. In 1988, the BSSR began utilizing the Cheneau brace in place of the Milwaukee and Boston braces because, at least theoretically, the Cheneau would produce the necessary detorsional forces with no deleterious effect on the sagittal configuration of the spine. The main impetus for such a change was the intention to prevent the flatback syndrome so often associated with the Milwaukee and Boston braces. A secondary justification was to find a better correlation between the principles of correction applied in physical therapy and bracing. The Chêneau brace was the closest to this correlation, in spite of the fact that initially we observed failures in the Chêneau original classification with cases where the basic three curves or four curves brace design produced undesired changes in the original curve pattern or resulted in inadequate in-brace corrections. We also noted much confusion and poor subjective reliability among orthopaedic technicians using Chêneau principles. During the 1990's, the King classification was adopted by some teams using the Chêneau brace. In order to be consistent with the terminology used by Chêneau, the term 'non three-non four' for the King Type III was adopted as his category did not fit clearly with the definition of any of the basic types. In contraposition, King I was considered 'four curve pattern', Type IV and V were considered 'three curve pattern', and while King II generated some doubts in us, it was always treated as 'four curve pattern' by Chêneau himself. We noted the poor reliability of the King classification early on and considered the Lenke classification as soon it was published. Although de Mauroy and collaborators [18] have proposed technical specifications in brace construction according to the different Lenke types, this classification appears to be unnecessarily complex in decision-making about the right design when using the Chêneau brace and its derivatives in the elected treatment. The first author of this paper (MR) developed the first classification to correlate curve pattern and brace design [19]. This first classification was based on SRS nomenclature, with some similarities with Coonrad [20] and showed good intraobserver reliability although, in unpublished results, Weiss found a poor interobserver reliability. With simplicity in mind, in the second classification we have combined clinical and radiological criteria in order make brace design more logical. Clinical criteria are, in part, those described previously by Lehnert-Schroth, while radiological criteria are new. In this paper we present this new classification. The main purpose of the present study is to estimate intra- and inter-observer reliability of the Rigo Classification in its radiological aspect.

## Methods

### The Rigo Classification

The classification has been developed in order to define specific principles of correction when treating with a particular type of brace, namely the Chêneau brace and its derivatives. The Chêneau principles of correction have been redefined by Rigo (Rigo System Chêneau - RSC) using biomechanical descriptions instead of the old anatomical description made by Jacques Chêneau. The brace provides detorsional forces by combining the following biomechanical principles: Translation and three-point pressure system/s in the frontal plane, pair/s of forces in the transversal plane, physiological profile and sagittal alignment. The pressure or contact areas, also called pads, are designed with a specific shape, level and direction for correction in the frontal plane and at the same time in the transversal plane by forming pairs of forces for derotation. A more thorough description of the biomechanical principles of the brace has already been published [21], and is also offered in additional file 1 (also see additional file figures 2, 3, 4, 5, 6, 7, 8, 9, 10, 11, 12). Brace design correlates with the curve pattern in the frontal plane. The purpose of the classification is to offer a more accurate and reliable diagnosis in order to fabricate proper brace design.

**Figure 1:**
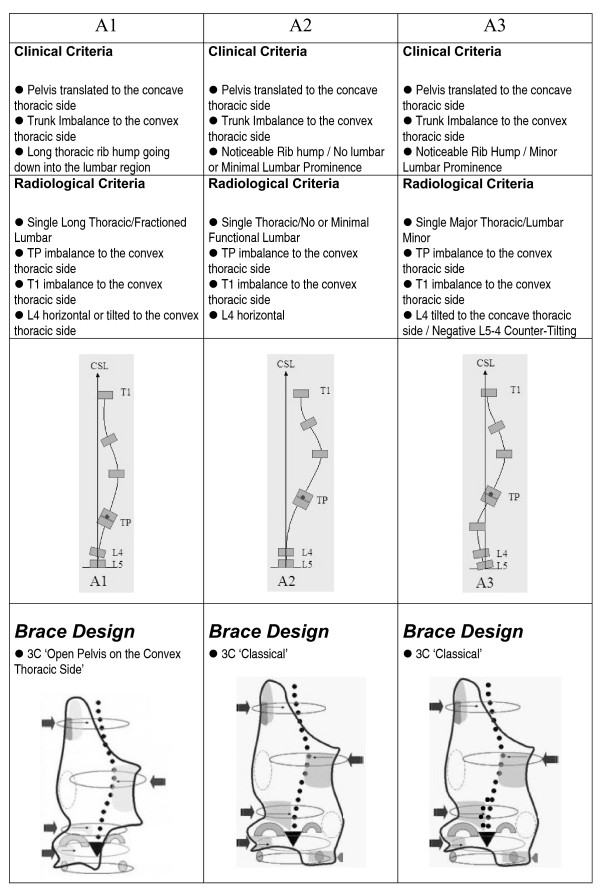
**Three-curve scoliosis pattern**. Clinical and radiological criteria for three-curve scoliosis pattern. Different sub-types can be defined according to those criteria. Specific brace design can be seen for each sub-type.

**Figure 2:**
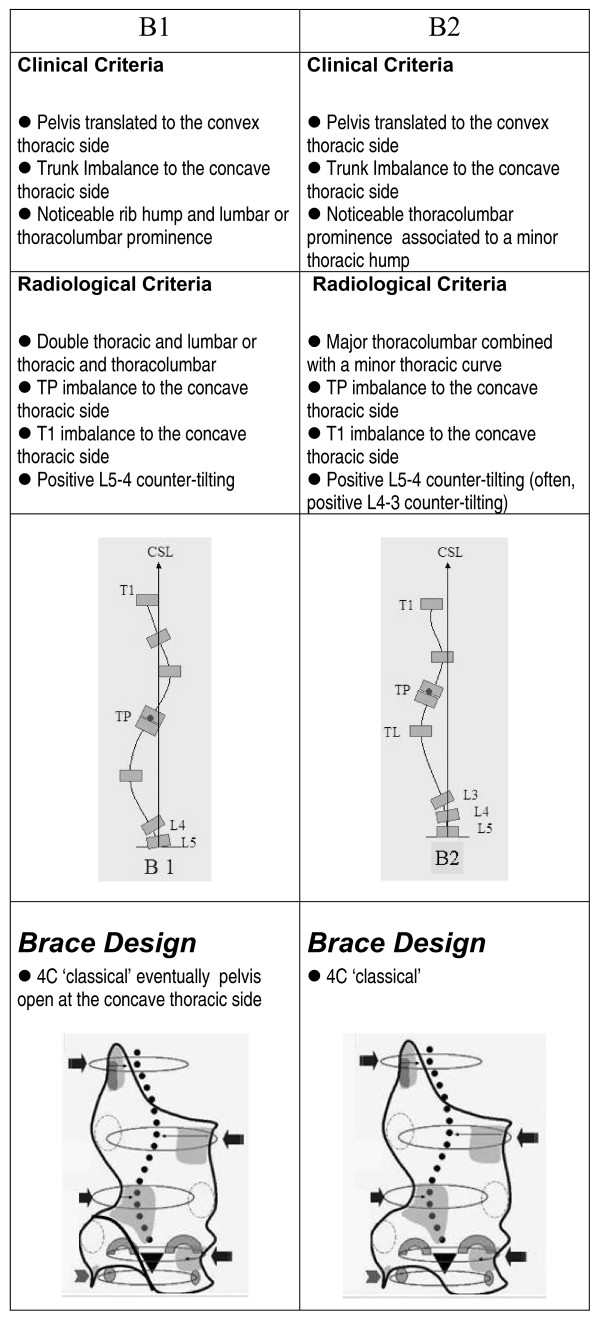
**Four-curve scoliosis pattern**. Clinical and radiological criteria for four-curve scoliosis pattern. Different sub-types can be defined according to those criteria. Specific brace design can be seen for each sub-type.

**Figure 3:**
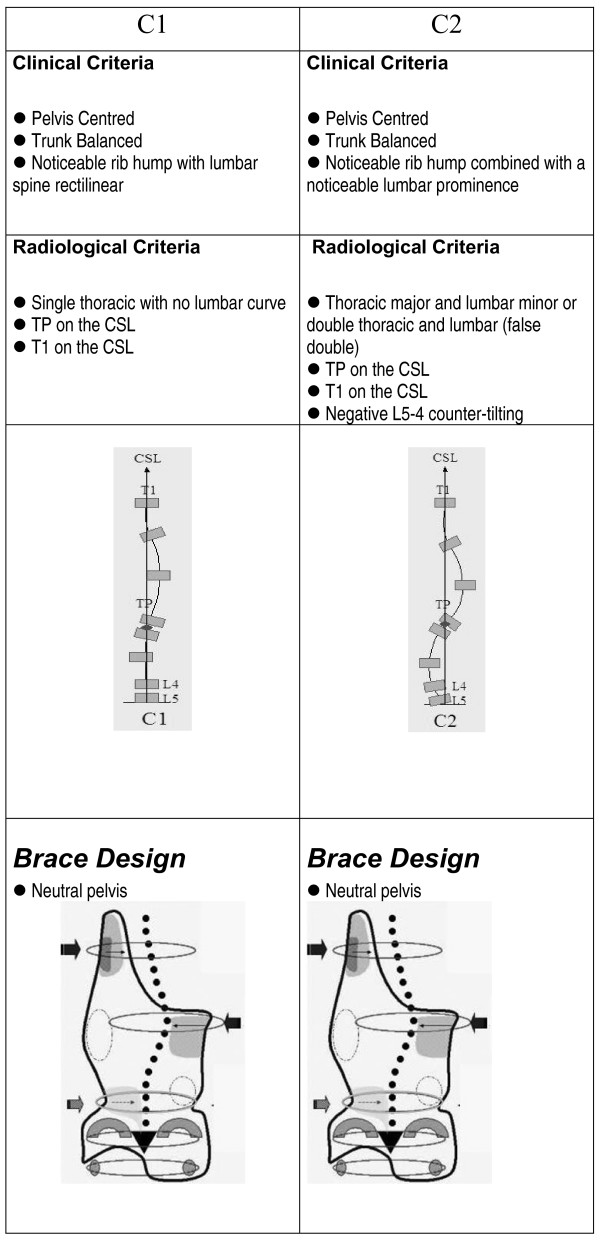
**Non three- Non four scoliosis curve pattern**. Clinical and radiological criteria for four-curve scoliosis pattern. Different sub-types can be defined according to those criteria. Specific brace design can be seen for each sub-type.

**Figure 4:**
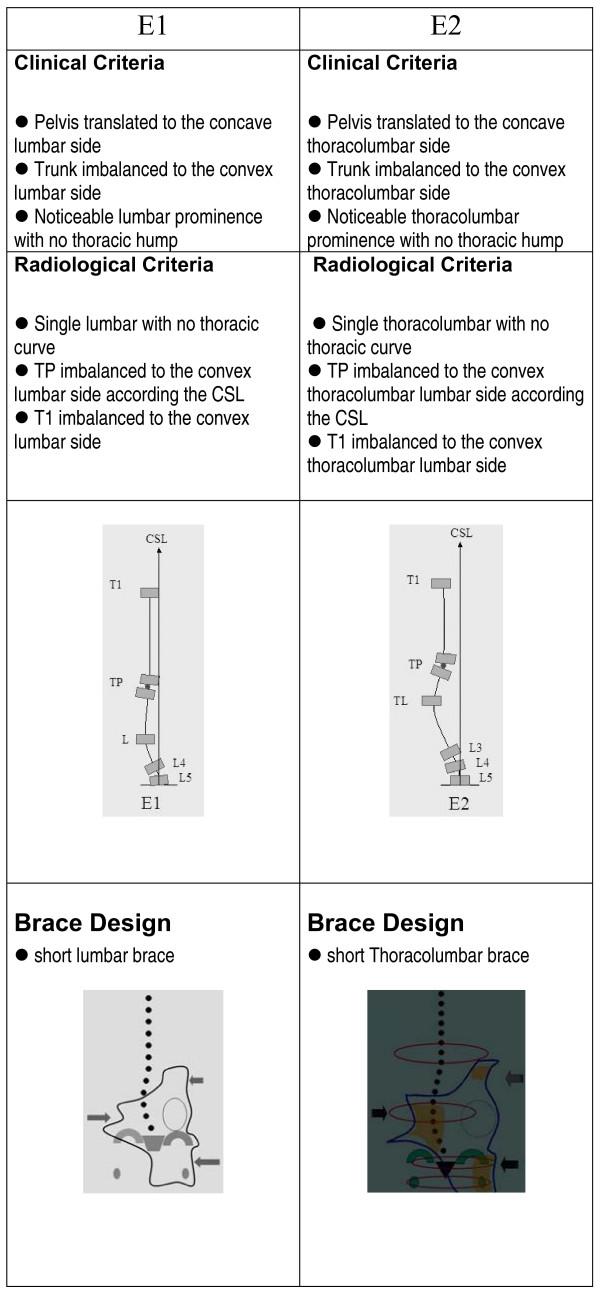
**Single lumbar/thoracolumbar curve pattern**. Clinical and radiological criteria for single lumbar and thoracolumbar scoliosis pattern. Different sub-types can be defined according to those criteria. Specific brace design can be seen for each sub-type.

The clinical and radiological criteria for the classification, curve pattern and blueprint of the brace design are presented in figures 1, figure 2, figure 3, figure 4. A direct observation of the patient from dorsal view and in forward bending allows for an initial clinical diagnosis of four basic types called: (I)three curves, (II)four curves, (III)non three-non four and (IV)single lumbar or thoracolumbar. Following the clinical diagnosis, it is necessary to confirm the type by using a radiological frontal projection and when necessary to choose a particular sub-type. 'Three curves' basic type is divided in sub-types A1, A2 and A3 in relation with lumbar configuration. 'Four curves' basic type is divided in sub-types B1 and B2 in relation with thoracic configuration. Non three-non four basic type is divided in sub-types C1 and C2, also in relation with lumbar configuration. Single lumbar and thoracolumbar are also called E1 and E 2 respectively. 'D modifier' defines an upper thoracic structural curve, which can be present in any of the types A, B or C.

**Figure 5:**
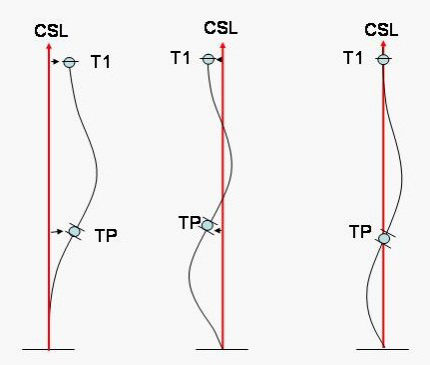
**Transitional Point Offset**. The Transitional Point ("TP") is defined as the middle point between the lower end vertebra (LEV) of the thoracic curve and the upper end vertebra (UEV) of the caudal curve. The position of the transitional point between the thoracic curve and any caudal curve according the Central Sacral Line ("CSL") defines 'transitional point offset'.

**Figure 6:**
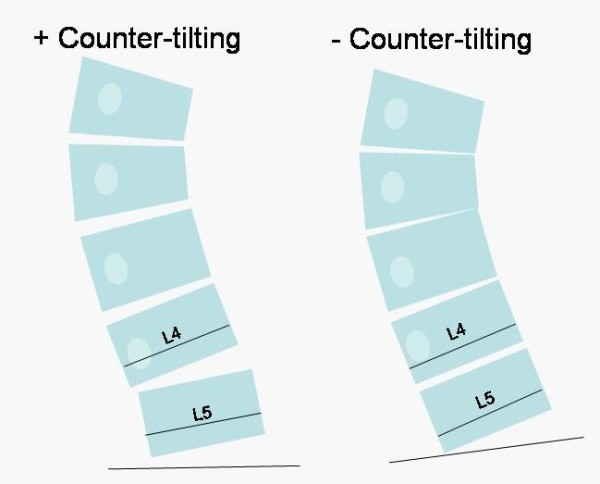
**L5-L4 Counter-tilting**. Caudal to L4, L5 can be tilted in the same direction and degree; this is defined as negative L5-4 counter-tilting. A positive L5-4 counter-tilting defines L5 tilted in the same direction as L4 but with lesser magnitude, forming a fractioned short lumbo-sacral curve. In other words, a negative L5-4 counter-tilting means that the lower end plate of L4 and the upper end plate of L5 are parallel; conversely, a positive L5-4 counter-tilting means that the upper end plate of L5 is clearly less tilted that the lower end plate of L4.

**Figure 7:**
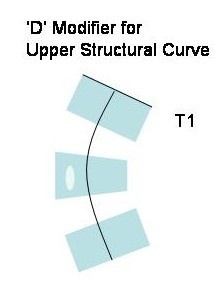
**D modifier**. When an upper thoracic structural curve is diagnosed a 'D modifier' will be recommended in the brace design.

The radiological criteria are based on:

1) A compatible curve pattern according the SRS terminology: single thoracic, single major thoracic, single long thoracic, compensatory lumbar, non-structural lumbar, minor lumbar, thoracic and lumbar double, thoracic and thoracolumbar double, single lumbar, single thoracolumbar and finally thoracolumbar major and thoracic minor. The apical level determines the name of the curve: T2-T11 for thoracic, T12 and L1 for thoracolumbar and L2 to L4 for lumbar.

2) The position of the transitional point between the thoracic curve and any caudal curve (compensatory lumbar, non-structural lumbar, minor lumbar, lumbar or thoracolumbar) according the Central Sacral Line ("CSL"). CSL is a vertical line representing the global axial axis [22], and it is drawn from the centre of the upper end plate of S1. The Transitional Point ("TP") is defined as the middle point between the lower end vertebra (LEV) of the thoracic curve and the upper end vertebra (UEV) of the caudal curve. Thus, when these two vertebrae are different, for example T12 and L1, the TP is located in the centre of the inter-vertebral disc T12-L1. When there is a neutral vertebra which acts as LEV of the thoracic curve and at the same time as UEV of the caudal curve, the TP is located in the centre of this neutral vertebra. The transitional point can be balanced on the CSL or imbalanced (also called TP-offset) to the convex or to the concave thoracic side (figure 5).

3) The position of T1 according to the CSL. T1 can be balanced on the CSL or imbalanced (also called T1-offset) to the convex or to the concave thoracic side.

4) The orientation of L4 in the frontal plane and its relation with L5. L4 can be, a) horizontal or b) tilted (i.e. frontal rotation) to the convex or to the concave thoracic side. In presence of a lumbar curve, no matter whether the curve is non-structural or structural, L4 will be tilted to the concave thoracic side. Caudal to L4, L5 can be tilted in the same direction and degree; this is defined as negative L5-4 counter-tilting. A positive L5-4 counter-tilting defines L5 tilted in the same direction as L4 but with lesser magnitude, forming a compensatory incomplete lumbo-sacral curve. In other words, a negative L5-4 counter-tilting means that the lower end plate of L4 and the upper end plate of L5 are parallel; conversely, a positive L5-4 counter-tilting means that the upper end plate of L5 is clearly less tilted that the lower end plate of L4. When the caudal curve is thoracolumbar rather than lumbar, the positive counter-tilting can be located at both levels L4-3 and L5-4 (Figure 6).

5) We have observed that any of the curve patterns can be combined with a primary or a secondary (to a previous brace treatment) upper thoracic structural curve. We have called this 'D' modifier (Figure 7). According to the Moe and Kettleson classification modified by Lonstein, the upper structural curve could be single or combined with a main thoracic structural curve (thoracic double major) but some authors have also described the combination of upper structural, main thoracic structural and lumbar or thoracolumbar structural using the term 'triple structural'.

### Reliability study

The study was designed to test the intra-observer and the inter-observer reliability of the radiological criteria. Three different observers, one medical doctor (MR), one physiotherapist (MV) and one orthotist (DG) were asked to classify a set of 51 consecutive AP radiographs taken from the database, including all the scoliosis types. The main author (MR) had to classify the whole set for a second time 78 hours after the first trial. The X-rays were presented to each observer in a D4 printed version with no pre-determined marks. For the intra-observer study the whole set of X-rays was printed twice and ordered in a different way.

Inter-observer Kappa values were calculated three times (MR versus MV, MV versus DG and MR versus DG), as well as the mean value, with an acceptance of >0.70. Intra-observer Kappa value was calculated (MR versus MR) with an acceptance of >0.70. Considering the correlation between type and brace design we defined six categories: A1 type, A2 + A3 types, B1 + B2 types, C1 + C2 types, E1 and E2. The upper structural curve was not considered because the need to use the 'D modifier' in the brace design is closely associated with the presence of an upper rib hump observed during the direct examination of the patient in forward bending.

## Results

Results are shown in Table 1, Table 2, Table 3, Table 4. The mean Kappa value in the inter-observer test was 0.71 (range 0.61-0.81). The Kappa value in the intra-observer test was 0.87. Both inter- and intra-observer mean Kappa values were over the acceptance value of 0.70. The highest intra- and inter-observer agreement was noted in types B1 + B2, E1 and E2. Thus, the so called 'four curves' scoliosis pattern or 'true double' as well as single lumbar and thoracolumbar are easy to diagnose by using these radiological criteria. Agreement in diagnose of the A and C types is less and the reasons are further discussed.

**Table 1:** Inter-observer Kappa coefficient (MR versus MV)

	A1	A2-3	B1-2	C1-2	E1	E2	
A1	**2**	0	0	1	0	0	3/51

A2-3	1	**7**	0	1	0	0	9/51

B1-2	0	0	**15**	0	0	1	16/51

C1-2	0	1	2	**12**	0	0	15/51

E1	0	0	0	0	**1**	0	1/51

E2	0	0	0	0	0	**7**	7/51

	3/51	8/51	17/51	14/51	1/51	8/51	

Kappa coefficient = 0.81

**Table 2:** Inter-observer Kappa coefficient (MV versus DG)

	A1	A2-3	B1-2	C1-2	E1	E2	
A1	**2**	1	0	0	0	0	3/51

A2-3	1	**7**	0	1	0	0	9/51

B1-2	0	0	**14**	2	0	0	16/51

C1-2	0	2	2	**10**	0	0	14/51

E1	0	0	0	0	**1**	0	1/51

E2	0	0	3	0	0	**5**	8/51

	3/51	10/51	19/51	13/51	1/51	5/51	39/51

Kappa coefficient = 0.71

**Table 3:** Inter-observer Kappa coefficient (MR versus DG)

	A1	A2-3	B1-2	C1-2	E1	E2	
A1	**1**	2	0	0	0	0	3/51

A2-3	2	**6**	0	1	0	0	9/51

B1-2	0	0	**13**	3	0	0	16/51

C1-2	0	1	4	**10**	0	0	15/51

E1	0	0	0	0	**1**	0	1/51

E2	0	0	2	0	0	**5**	7/51

	3/51	9/51	19/51	14/51	1/51	5/51	36/51

Kappa coefficient = 0.61

**Table 4:** Intra-observer Kappa coefficient (MR versus MR)

	A1	A2-3	B1-2	C1-2	E1	E2	
A1	**3**	0	0	0	0	0	3/51

A2-3	1	**7**	0	1	0	0	9/51

B1-2	0	0	**15**	1	0	0	16/51

C1-2	0	0	2	**13**	0	0	15/51

E1	0	0	0	0	**1**	0	1/51

E2	0	0	0	0	0	**7**	7/51

	3/51	7/51	17/51	15/51	1/51	7/51	46/51

Kappa coefficient = 0.87

## Discussion

The present study demonstrates a good inter-observer reliability as well as intra-observer reliability regarding the radiological criteria of this new classification. Although in one out of three inter-observer tests (MR vs DG) the Kappa value was found to be lower than the acceptance value, the mean value was slightly superior to this (>0.70). Considering several factors we have initially defined such reliability as fair. First at all, the size and printed quality of the X-rays were not optimal. We decided to use the present method just for practical reasons because one of the observers was located remotely and printed versions were more easily shared. On the other hand, the radiological criteria were defined to confirm the first clinical diagnosis. Thus, during the daily clinical practice the radiological classification is seldom used in isolation to make the decision about the brace design. A protocol has been clearly established where the patient has to be observed and examined in order to make the initial diagnosis choosing one out of the four basic categories: three curves, four curves, non three-non four and lumbar or thoracolumbar. Afterwards, we recommend marking the CSL, the TP, T1, the lower end plate of L4 and the upper end plate of L5 on the X-rays in order to confirm the first diagnostic impression. The differential diagnosis between 'three curves' and 'four curves' is easy to make. However 'non three-non four' is sometimes difficult to classify when using just radiological criteria because a patient diagnosed clinically as 'non three-non four' could present a slight but insignificant offset of the transitional point. Unfortunately, we have not been able to establish a numerical value for the degree of offset necessary to confirm whether a scoliosis is 'three' or 'four' versus 'non three-non four'. Following the protocol, once we have clinically diagnosed a particular case as 'non three-non four', most commonly we will observe on the X-ray that TP is exactly on the CSL or with an insignificant offset. Although it is a subjective method, it is easily implemented with minimum experience. Notwithstanding, the authors are now working out a more accurate and objective method to define clinical balance at the transitional point. It has been argued that some terms used in this classification are not worldwide accepted and this creates confusion. Contrary, this paper endeavours to clarify this topic by establishing parallelisms between the different terms and types defined in several classifications. The terms 'three curves' and 'four curves' result exotic to many colleagues, however theses are familiar terms used for a long time by physiotherapists, orthotist and doctors following the European-German school. However, our current recommendation to those following such European-German school is to use a world wide SRS terminology like for example that proposed by Moe, Kettleson and Lonstein and later classify according the introduced terminology A, B, C, D and E in order to give physiotherapy and brace specifications for the Chêneau type brace and its derivates.

Consequently, the present study demonstrates a good inter-observer reliability regarding the radiological criteria of the classification. Intra-observer reliability was also fair and the experience of the first author is that intra-observer reliability would be close to 1 when considering both clinical and radiological criteria, although this needs further studies. A limitation of the present study is the small number of participants in the inter-observer test which reduces the ability to perform rigorous statistical analysis. This is a first trial conducted as a preliminary study with the minimum participation of one doctor, one physiotherapist and one orthotist familiar with the classification and terminology. As a result of this pilot study we have re-defined some criteria in order to improve clarity. Further studies will be necessary to test the radiological criteria after revision as well as the whole classification system with participation of doctors who were not involved in the development of the classification. Another question is relative importance of all criteria in the decision making process. This was not addressed in our present study, but will be analysed in the future. From experience in the development of those criteria, the curve pattern and the transitional point offset have the highest consistency. The negative L5-4 counter-tilting in A3, C1 and C2 has sometimes been found to be a false positive due to a leg length discrepancy, but in those cases, during the in-vivo examination of the patient, the pelvis is found to be coupled to the lumbar curve systematically. The pelvis is uncoupled to the lumbar region throughout a compensatory lumbo-sacral curve just in four curve scoliosis pattern and this is easily appreciated when a minimally experienced clinician directly examines the patient. No T1 offset or even T1 offset to the unexpected side was found in some A2 and A3 cases, mostly in patients previously treated with a brace. Some 'non three-non four' cases are also associated with a something more than insignificant offset at T1, especially when there is a structural upper curve or when the thoracic scoliosis is asymmetric (major inclination in the upper end vertebra in comparison with a minor inclination in the lower end vertebra).

**Figure 8:**
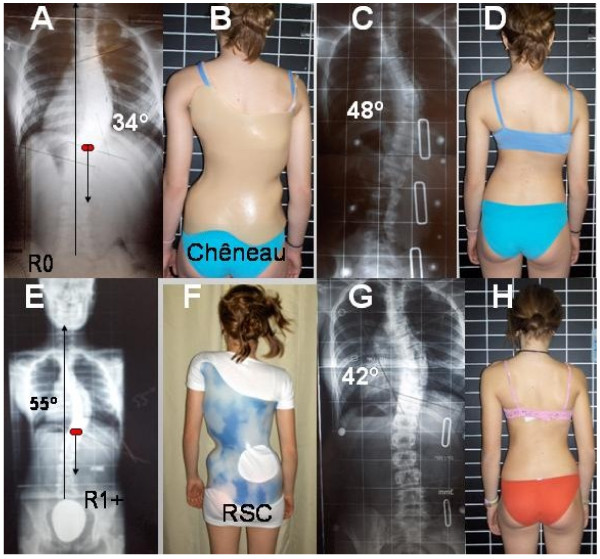
**Wrong and right brace design according to curve pattern**. A 11-year old girl with scoliosis of 34° Cobb (A), treated with a four-curve scoliosis brace (B), progressing to 48° (C) in brace and 55° (E) out of brace after one year of treatment (D). A brace well designed according to her classification of a three curves- A2 type (F) produced an acceptable in brace correction (G) with a good clinical response (H). She had criteria to be diagnosed as a three curves pattern from the first X-Ray.

The importance of the present study has to be considered from the perspective of standardisation in bracing when the Chêneau brace or any of its derivatives is the chosen treatment. The Chêneau brace is one of the most frequently used braces for the treatment of AIS in many European countries but its standard is poor. This is also the reason to use the term Chêneau derivatives. Chêneau based his treatment principles on anatomical descriptions rather than on biomechanical principles and he proposed a simple classification which, historically, has created some confusion. An example of a treatment error produced by the wrong brace design after a bad classification can be seen in figure 8. A girl with a right thoracic scoliosis measuring 34° Cobb (A) started treatment with a 'Cheneau' brace built following the 'four curve scoliosis' principles of correction (B). The curve was observed to progress to 48° in the brace(C). The treatment team considered the girl as a firm candidate for surgery because of her bad response to bracing. They did not consider that the improper brace was prescribed. One year after treatment initiation she progressed to 55° out of brace (E). Clinically she had to be clearly classified as 'three curve scoliosis pattern' (D). Radiological criteria also fitted with A2 type (E) and her brace had to be constructed following 'three curve scoliosis principles (F). A 'classic three curve scoliosis brace' reduced the scoliosis from 55° to 42° (G). In-brace correction can be defined as acceptable in the second brace, considering that she had been wearing a first brace with a deficient design for one year, probably making the curve highly structural. The result is acceptable especially when looking at her clinical aspect after just a few months treatment with the correct brace design (H). A treatment failure like this could easily be prevented with a correct initial diagnosis, classification and brace design.

## Conclusions

A new scoliosis classification has been described correlating with brace treatment. The classification employs clinical and radiological criteria. The radiological criteria have shown a fair intra- and inter-observer reliability when used by clinicians familiar with the Rigo Classification. The optimization in diagnosis, classification and brace design will likely reduce treatment failures.

## Consent

Written consent for publication of case report and photos was obtained from the mother of the girl showed in figure number 8.

## Competing interests

The authors declare that they have no competing interests.

## Authors' contributions

MR conceived of and described the new classification, conceived of the study, defined the design, participates in the reliability study and performed the statistical analysis. He also prepared and approved the final manuscript.

DG and MV participated in the design and coordination of the study and helped to draft the manuscript. They participated in the reliability study, read and approved the final manuscript.

## Supplementary Material

Additional file 1 **Biomechanical principles of the Rigo-System-Chêneau Brace (RSC-Brace)**. Short paper describing the principles of correction of the so called Rigo-System-Chêneau Brace (a Chêneau derivate). The paper is supported by a set of figures submitted as Additional file 2, Additional file 3, Additional file 4, Additional file 5, Additional file 6, Additional file 7, Additional file 8, Additional file 9, Additional file 10, Additional file 11, Additional file 12. Click here for file

Additional file 2 **Detorsional forces (figure)**. The brace derotates the thoracic region (b) against the lumbar region (a), with a counter-rotation pad pushing to ventral on the upper thoracic region. Derotation of one region against another region produces detorsional forces. Click here for file

Additional file 3 **Three-point-pressure system (figure)**. Different three-point-pressure systems correct in the frontal plane. The thoracic concavity has to be decollapsed (mirror effect) to allow derotation. A ventral pad works in combination with a dorsal pad to form a 'pair of forces' for derotation at the main thoracic region. Click here for file

Additional file 4 **Sagittal profile and alignment. Local derotation and correction of the structural flat back (figure)**. Alignment and physiological sagittal profile to normalize the sagittal geometry of the spine. Correction of the structural flat back at the main thoracic region is related to breathing mechanics promoted by the specific design of the brace in the transversal plane. A 'pair of forces' for derotation acts at the apical level of the main thoracic curve. The orientation of the dorsal pad is more sagittal in comparison with the orientation of the ventral pad. This specific design makes the ventral pad to created the major force for derotation. The apical vertebra moves backwards coupled to the concave thoracic ribs. Click here for file

Additional file 5 **Blueprint of the A1 type brace (figure)**. A1 type brace design. A single three-point-pressure system corrects high efficiently the long thoracic curve. The brace does not cover the pelvis on the convex thoracic side. Pelvis is over-corrected. Click here for file

Additional file 6 **Blueprint of the A 2 and 3 type braces (figure)**. The main three-point-pressure system is like in A1 type and corrects the main thoracic curve. A secondary three-point-pressure system, with a counter-trochanter pad corrects the lumbar curve. Lumbo-pelvic region is overcorrected. Click here for file

Additional file 7 **Blueprint of the B type brace - classic design- (figure)**. Two main three-point-pressure systems correct the structural lumbar or thoracolumbar curve and the thoracic curve. A secondary three-point-pressure system, with a counter-trochanter pad on the concave thoracic side, will correct the compensatory lumbo-sacral curve. The lumbar or thoracolumbar pad can be wide (higher apex) or narrow (lower apex). Pelvis is over-corrected. Click here for file

Additional file 8 **Blueprint of the B type brace - open (figure)**. The B type brace can be built with no trochanter counter-pad (open pelvis model). In the picture the short lumbar pad has been designed for a B1 type. B2 use to be built with a wider pad. Click here for file

Additional file 9 **Blueprint of the C type brace (figure)**. A single three-point-pressure system (lumbar-thoracic-upper thoracic) corrects the main thoracic curve. A secondary system corrects the lumbar curve or prevents a lumbar curve to be created. Pelvis is neutral. Click here for file

Additional file 10 **Comparison of brace types B and C at the lumbo-pelvic region (figure)**. In brace type B, lumbar region and pelvis are translated one against the other. In brace type, pelvis remains neutral and a lumbar pad corrects a lumbar curve or prevents that a lumbar curve is secondarily created. Click here for file

Additional file 11 **Blueprint of the E type brace (figure)**. A single three-point-pressure system corrects the lumbar or thoracolumbar single curve. Pelvis and lumbar (or thoracolumbar) regions are translated one against the other with a counter-thoracic pad pushing caudally to the virtual thoracic apex. Click here for file

Additional file 12 **A brace with 'D modifier' shape at the upper thoracic region (figure)**. The upper structural curve can be treated with a specific brace design. This is an example of A2 -3 type brace with the D modifier for an upper structural left curve. Click here for file
