# Supplementary material for: A specific scoliosis classification correlating with brace treatment: description and reliability
Source: Scoliosis. 2010 Jan 27;5:1. doi: 10.1186/1748-7161-5-1 (PMC2825498; doi:10.1186/1748-7161-5-1)
Supplement: Additional file 1 — Biomechanical principles of the Rigo-System-Chêneau Brace (RSC-Brace). Short paper describing the principles of correction of the so called Rigo-System-Chêneau Brace (a Chêneau derivate). The paper is supported by a set of figures submitted as Additional file 2, Additional file 3, Additional file 4, Additional file 5, Additional file 6, Additional file 7, Additional file 8, Additional file 9, Additional file 10, Additional file 11, Additional file 12. [file 1748-7161-5-1-S1.DOC]

**Biomechanical principles of the Rigo-System-Chêneau Brace (RSC-Brace)**

Introduction

The Chêneau brace is defined as a thermoplastic brace molded on a hyper corrected positive plaster model. The corrective pads are not added into a symmetric plastic cylinder, as in the Boston brace technique, but designed directly into the positive model. Shape, depth and orientation of the pads are specifically built to act on the convex areas of the deformed trunk. The brace design appears radical, but that comes from the large expansion spaces rather than from the pads. The expansion spaces make hypercorrection possible by allowing the patient to move, to breathe and to grow towards the open spaces. Chêneau outlined the rules to correct the positive model in several books and papers [1, 2]. However, Chêneau has enunciated his principles in terms of anatomical observations rather than biomechanical principles. The author (MR) has developed a correction model based on biomechanical descriptions (Rigo-System-Chêneau or RSC principles) [3]. The RSC will provide the necessary passive forces by means of highly selective pads which produce several corrective systems.

However, the so called ‘expansion rooms” are more than just ‘windows’ and therefore are providing correction mechanisms. The different correction systems provided by pads in combination with expansion rooms facilitates the breathing mechanics. The corrective forces produced by the breathing mechanics can be considered as dynamic forces. Such forces are hypothesized by providing the only effective mechanism able to fight against the thoracic structural flat back.

Mechanisms of correction

The general RSC principle can be enunciated as the ‘correction of the lateral curvature, axial rotation and structural flat back as well by means of detorsional forces’. Detorsion is produced by combining forces in the three planes of space: Derotation (transversal plane), deflection (frontal plane) and sagittal normalization (sagittal plane). The RSC provides different correction systems to accomplish detorsion:

1. ‘Pair of forces’ systems to derotate in the transversal plane;
2. Three-point-pressure systems to increase correction in the frontal plane;
3. Physiological sagittal alignment and profile.

The pads and ‘expansion rooms’ are both built on a positive plaster mould of the patient following a well planed design. There are different designs according to the curve pattern. The curve pattern is defined by using the new Rigo Classification: A (1, 2, and 3), B (1 and 2), C (1 and 2) and E (1, 2); D modifier means a specific design for an upper thoracic structural curve. By extension the basic brace model can be called A, B, C or E. This terminology is currently recommended to substitute the antiquated terminology of ‘three curves’, ‘four curves’, ‘non 3 – non 4’, lumbar and thoracolumbar, which created some confusion among those more familiar with SRS terminology and world wide spread classifications (Moe and Kettleson, King and Lenke classifications). This topic is discussed in the original paper presenting this new classification.

The pads are basically defined as ‘dorsal pads’ and ‘ventral pads’. A ‘*dorsal* pad’ works always in association with a ‘*ventral* pad’ in order to form a ‘pair of forces’ for a trunk regional derotation. A trunk region is defined as the part of the trunk affected by any particular scoliotic curve. Detorsional forces are produced when a trunk region is derotated against two adjacent regions located *caudally* and *cranially* (additional file 2). Any defined ‘pair of forces’ has to work at the same level or height; otherwise the effect will be different than derotation. Every ‘*dorsal*’ and ‘*ventral* pad’ has a *lateral* to *medial* component which depends on the orientation of the pad. Consequently, the ‘dorsal’ and ‘ventral pads’ form different three-point-pressure systems. Thus, pads are shaped on the positive mould at a specific level as a part of a particular three-point-pressure system. Any three-point-pressure system is built to correct a scoliotic curve in the frontal plane. Correction in the frontal plane will decollapse the concave region making derotation possible (additional file 3). The brace should present a physiological sagittal alignment and profile to normalize the sagittal configuration of the spine. But the same way the structural deformity affects the frontal and transversal plane it affects the sagittal plane. A major or minor structural flat back is observed in the thoracic region in most of the thoracic scoliosis as well as in the thoracic component of a double scoliosis. It is not possible to reconstruct the normal profile of the spine in the sagittal plane but only on those cases where correctibility is very high due to a minor structural component in combination with the flexibility of the soft elements. This is the same way the scoliotic frontal curve can be corrected or overcorrected in very few cases due to these same conditions.

More commonly, it would remain uncorrected in a certain amount of structural flat backs. The combination of pads with expansion rooms promoted corrective breathing mechanics to fight against the remaining structural flat back in the thoracic region (additional file 4). This is an exclusive particularity of the brace originally described by J. Chêneau.

Design according to curve pattern

Brace type A:

A1: Additional file 5 shows the only three-point-pressure system for this particular curve pattern. The main thoracic curve is a long curve going down into the lumbar region. There is a three-point-pressure system working on three defined regions: 1) the pelvis coupled to the low lumbar region; 2) a long thoracic region which includes the upper lumbar region and 3) the upper thoracic region. The three – point – pressure system works here highly efficient due to the long level arms. In brace corrections with this brace model A1 is the highest (4). The pads built to form the three – point – pressure system are oriented, in a way that they act simultaneously as pair of forces for derotation, which is the rule for all the below described types.

A2 and A3: Additional file 6 shows the two ‘three – point – pressure systems’ designed for these two similar curve patterns. The pelvis is coupled with lumbar region so the main three – point – pressure system works similar than in Type A1 but with a shorter thoracic pad in the cranial-caudal direction. On the other hand, the lumbar concavity requires a secondary three – points system formed by the main thoracic pad, the lumbo-pelvic pad and a counter – trochanter pad.

From the technical point of view the correction of a mould in patients with curve type A is that lumbo-pelvic and thoracic regions are over-corrected one against the other in the frontal plane.

Brace type B:

Additional file 7 shows the three ‘three – point – pressure systems’ built for these two curve patterns. One of the two main systems work to correct in the frontal plane of the lumbar or low thoracolumbar curve (B1) or the high thoracolumbar curve (B2) and is formed by three pads located at the lumbar or thoracolumbar convexity, the thoracic convexity and the pelvic convexity (same side than of the thoracic convexity). The second of the two main systems work to correct the thoracic curve and is formed by a convex thoracic pad in combination with the lumbar or thoracolumbar pad and an upper thoracic pad. In this particular curve pattern, the pelvis and lumbar regions are uncoupled. These two regions have to be over-corrected in the frontal plane by translating one against the other. Sometimes translation between the two regions is enough for correction and postural balance in the upright position (the pelvis can be partially opened on the convex lumbar or thoracolumbar side, as seen in additional file 8) but many times is necessary to build a secondary three – point – pressure system to decollapse the lumbosacral concavity. In this last case it is necessary to design a counter- trochanter pad on the convex lumbar or thoracolumbar side.

Brace type C

In this particular curve pattern pelvis is, like in type A, coupled to the lumbar region but there is no need to over-correct in the frontal plane by translating lumbo-pelvic region and thoracic regions one against the other. When correcting the positive mould, lumbo- pelvic region are taken as neutral region and kept in its neutral position. Cranially, a single three – point – pressure system is built with a lumbar pad, a main thoracic pad and an upper thoracic pad. Additional files 9 and 10 show the design for this curve pattern.

Brace type E

E1 and E2 also require a single three –point – pressure system established like shown in additional file 11. The E type brace is a three point derotation-deflection brace with a short profile. Pelvis is partially opened on the lumbar or thoracolumbar convex side in most of patients with this curve pattern. Sometimes, a secondary three – point – pressure system is required to decollapse the lumbosacral concavity like in type B when trunk balance can not be achieved in the frontal plane with the short three – points brace.

The D modifier

This is related to the presence of a structural upper thoracic curve. The upper thoracic section is built with a specific shape and orientation. The pad is rounder, with a more anatomical shape in comparison with the classic one, pushing towards the medial and does not go as high as in the classical design. Additional file 12 shows this design.

Discussion

Biomechanical principles of the RSC have been defined in this short paper. The correction of a positive mould to pass from the deformed scoliotic body shape to a corrected body shape prior to the fabrication of the plastic brace is a very difficult technique, which is achievable by few orthopedic technicians.

The technician should perfectly know the three-dimensional correction in any particular curve pattern.

Significant experience, education and learning curve are required to master this technique. This is most likely the reason for a bad standard observed when the classical Chêneau brace is prescribed. Variability in the construction of the classical Chêneau brace is very high. In order to improve standards, a CAD CAM system was created during the second half of the past decade (1991-2000). This CAD CAM brace, called Ortholutions RSC brace which utilizes templates from a data base of more than 100 models corrected by the author of this paper (MR). The models can be modified according to patient specifications but keeping always the original correction design. In brace correction with the CAD CAM Ortholutions brace has been shown to be as good as that obtained with braces built from a handmade corrected mould (5).

# Acknowledgements

The authors wish to thank Grant Wood CPO, San Mateo, California, USA, for contributions in manuscript editing.

# References

1. Chêneau J: *Corset-Chêneau. Manuel d’orthopédie des scolioses suivant la technique originale.* Paris, Édition Frison-Roche; 1994
2. Weiss HR, Rigo M, Chêneau M: *Praxis der Chêneau Korsettversorgung.* Thieme, Stuttgart 2000.
3. Rigo M, Weiss HR: **The Chêneau concept of bracing- Biomechanical aspects.** *Studies in Health Technology and Informatics* 2008, **135**: 303-319
4. Rigo M, Gallo D: A new RSC brace design to treat single long thoracic scoliosis. In-brace correction with the new design compared to the classical design. SORORT meeting, Lyon, May 2009.
5. Gallo D: **Chancen und Möglichkeiten von CAD/CAM in der Orthopädie-Technik erläutert am Beispiel der RSC Korsett servicefertigung**. *Orthopädic-Technik* 2005, **10**: 704-711
