# Supplementary figures and images for: A specific scoliosis classification correlating with brace treatment: description and reliability
Source: Scoliosis. 2010 Jan 27;5:1. doi: 10.1186/1748-7161-5-1 (PMC2825498; doi:10.1186/1748-7161-5-1)

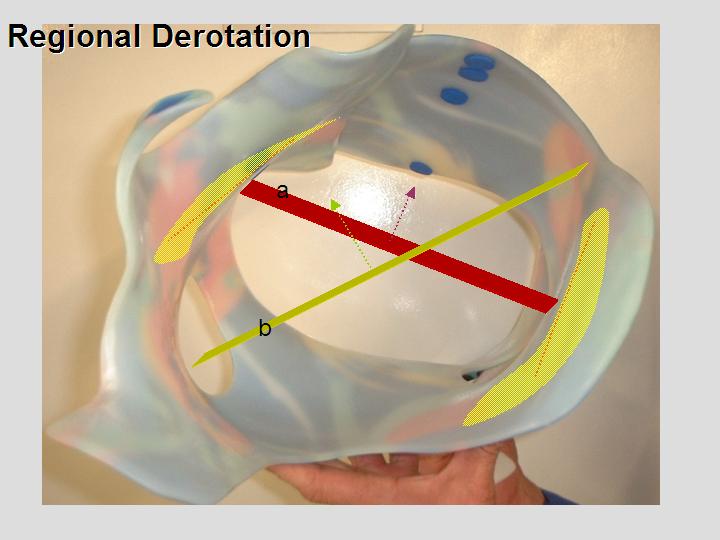

Supplement: Additional file 2 — Detorsional forces (figure). The brace derotates the thoracic region (b) against the lumbar region (a), with a counter-rotation pad pushing to ventral on the upper thoracic region. Derotation of one region against another region produces detorsional forces. [file 1748-7161-5-1-S2.JPEG]

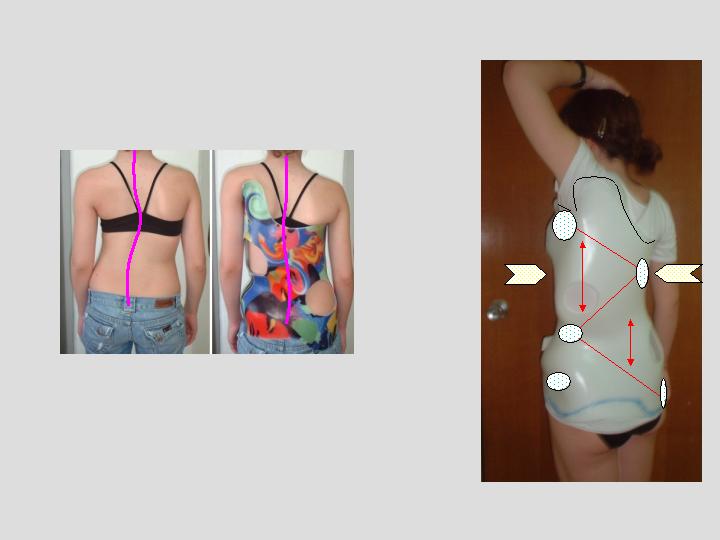

Supplement: Additional file 3 — Three-point-pressure system (figure). Different three-point-pressure systems correct in the frontal plane. The thoracic concavity has to be decollapsed (mirror effect) to allow derotation. A ventral pad works in combination with a dorsal pad to form a 'pair of forces' for derotation at the main thoracic region. [file 1748-7161-5-1-S3.JPEG]

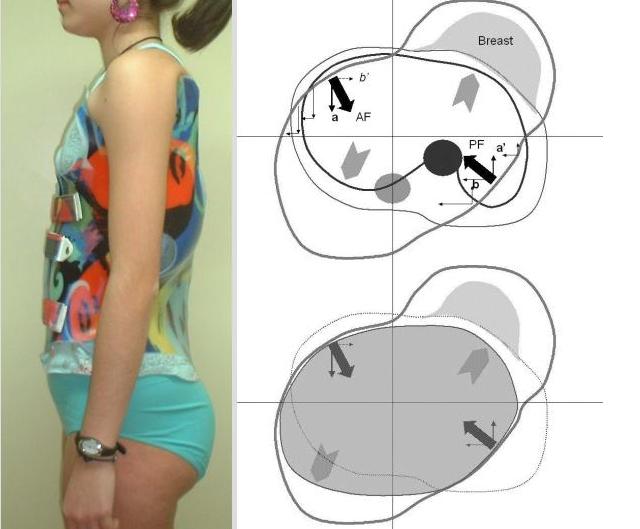

Supplement: Additional file 4 — Sagittal profile and alignment. Local derotation and correction of the structural flat back (figure). Alignment and physiological sagittal profile to normalize the sagittal geometry of the spine. Correction of the structural flat back at the main thoracic region is related to breathing mechanics promoted by the specific design of the brace in the transversal plane. A 'pair of forces' for derotation acts at the apical level of the main thoracic curve. The orientation of the dorsal pad is more sagittal in comparison with the orientation of the ventral pad. This specific design makes the ventral pad to created the major force for derotation. The apical vertebra moves backwards coupled to the concave thoracic ribs. [file 1748-7161-5-1-S4.JPEG]

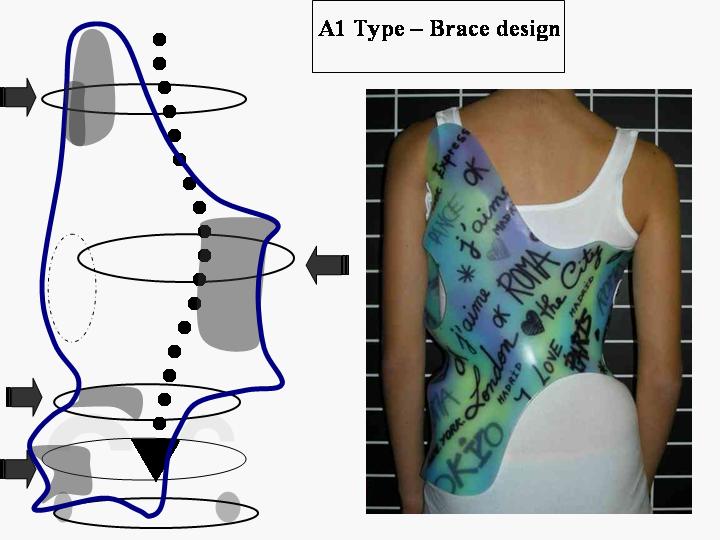

Supplement: Additional file 5 — Blueprint of the A1 type brace (figure). A1 type brace design. A single three-point-pressure system corrects high efficiently the long thoracic curve. The brace does not cover the pelvis on the convex thoracic side. Pelvis is over-corrected. [file 1748-7161-5-1-S5.JPEG]

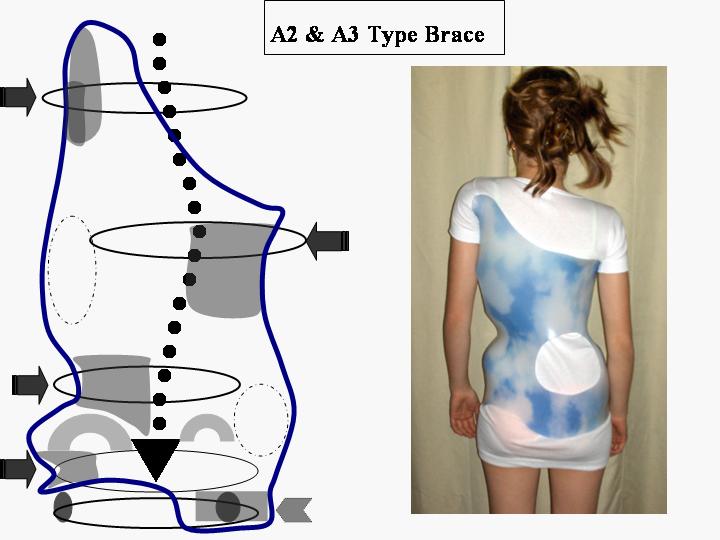

Supplement: Additional file 6 — Blueprint of the A 2 and 3 type braces (figure). The main three-point-pressure system is like in A1 type and corrects the main thoracic curve. A secondary three-point-pressure system, with a counter-trochanter pad corrects the lumbar curve. Lumbo-pelvic region is overcorrected. [file 1748-7161-5-1-S6.JPEG]

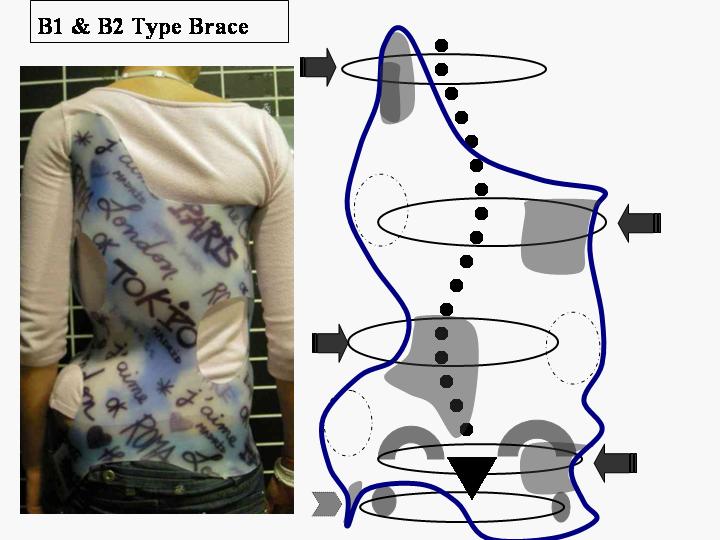

Supplement: Additional file 7 — Blueprint of the B type brace - classic design- (figure). Two main three-point-pressure systems correct the structural lumbar or thoracolumbar curve and the thoracic curve. A secondary three-point-pressure system, with a counter-trochanter pad on the concave thoracic side, will correct the compensatory lumbo-sacral curve. The lumbar or thoracolumbar pad can be wide (higher apex) or narrow (lower apex). Pelvis is over-corrected. [file 1748-7161-5-1-S7.JPEG]

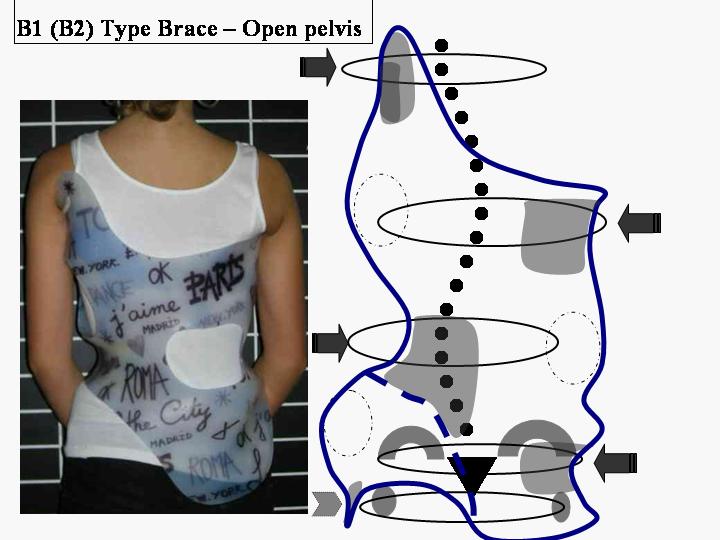

Supplement: Additional file 8 — Blueprint of the B type brace - open (figure). The B type brace can be built with no trochanter counter-pad (open pelvis model). In the picture the short lumbar pad has been designed for a B1 type. B2 use to be built with a wider pad. [file 1748-7161-5-1-S8.JPEG]

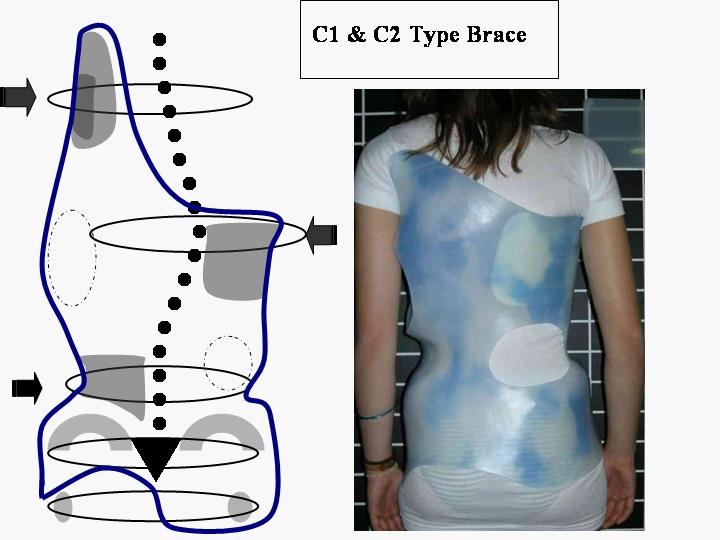

Supplement: Additional file 9 — Blueprint of the C type brace (figure). A single three-point-pressure system (lumbar-thoracic-upper thoracic) corrects the main thoracic curve. A secondary system corrects the lumbar curve or prevents a lumbar curve to be created. Pelvis is neutral. [file 1748-7161-5-1-S9.JPEG]

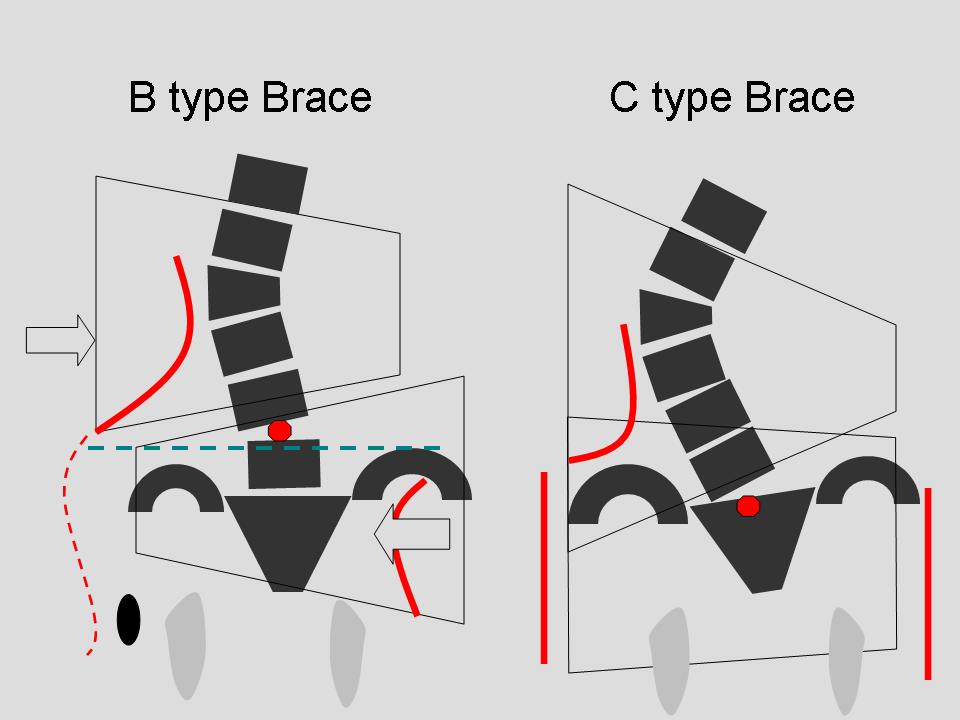

Supplement: Additional file 10 — Comparison of brace types B and C at the lumbo-pelvic region (figure). In brace type B, lumbar region and pelvis are translated one against the other. In brace type, pelvis remains neutral and a lumbar pad corrects a lumbar curve or prevents that a lumbar curve is secondarily created. [file 1748-7161-5-1-S10.JPEG]

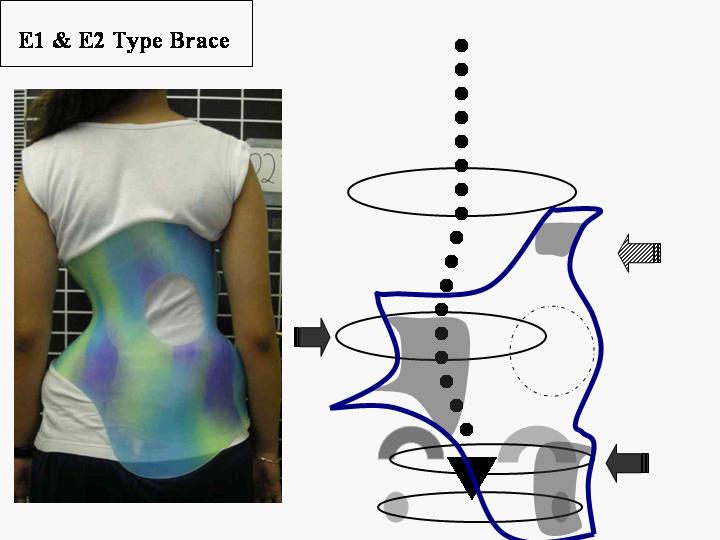

Supplement: Additional file 11 — Blueprint of the E type brace (figure). A single three-point-pressure system corrects the lumbar or thoracolumbar single curve. Pelvis and lumbar (or thoracolumbar) regions are translated one against the other with a counter-thoracic pad pushing caudally to the virtual thoracic apex. [file 1748-7161-5-1-S11.JPEG]

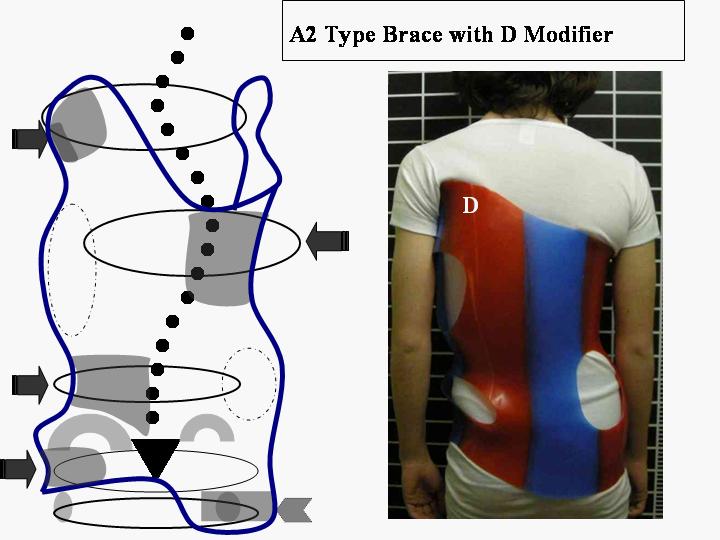

Supplement: Additional file 12 — A brace with 'D modifier' shape at the upper thoracic region (figure). The upper structural curve can be treated with a specific brace design. This is an example of A2 -3 type brace with the D modifier for an upper structural left curve. [file 1748-7161-5-1-S12.JPEG]
